# Epigenetics in neurodegenerative disorders induced by pesticides

**DOI:** 10.1186/s41021-021-00224-z

**Published:** 2021-12-10

**Authors:** Guangxia Yu, Qianqian Su, Yao Chen, Lingyan Wu, Siying Wu, Huangyuan Li

**Affiliations:** 1 Fujian Key Lab of Environmental Factors and Cancer, School of Public Health, Fujian Medical University, Fuzhou, Fujian Province China; 2 Department of Preventive Medicine, School of Public Health, Fujian Medical University, Fuzhou, Fujian Province China; 3 Key Lab of Environment and Health, School of Public Health, Fujian Medical University, Fuzhou, Fujian Province China; 4 Department of Epidemiology and Health Statistics, School of Public Health, Fujian Medical University, Fuzhou, Fujian Province China

**Keywords:** Neurodegenerative disease, Epigenetic, Nervous system, Environmental pollution, Pesticide, DNA methylation, Histone modifications, Non-coding RNA, RNA methylation, N6-methyladenosine

## Abstract

Neurodegenerative diseases are becoming major socio-economic burdens. However, most of them still have no effective treatment. Growing evidence indicates excess exposure to pesticides are involved in the development of various forms of neurodegenerative and neurological diseases through trigger epigenetic changes and inducing disruption of the epigenome. This review summaries studies on epigenetics alterations in nervous systems in relation to different kinds of pesticides, highlighting potential mechanism in the etiology, precision prevention and target therapy of various neurodegenerative diseases. In addition, the current gaps in research and future areas for study were also discussed.

## Introduction

With aging of the population in worldwide, neurodegenerative diseases, especially Alzheimer’s disease (AD) and Parkinson’s disease (PD) are becoming major socio-economic burdens. Their increasing prevalence and without effective treatment mean these diseases will be a challenge for future generations [1]. Although several cellular mechanisms and genes have been proved implicated in the onset and progression of the disease, the precise molecular underpinnings of these diseases remain unclear [2].

Epigenetics is generally defined as a heritable change in gene function, which can influence gene expression and subsequent protein expression levels without altering DNA sequence [3]. The epigenetic molecular factors include DNA methylation [4], histone modifications [5], non-coding RNA [6], chromatin structure [7], and RNA methylation [8]. Epigenetic dysregulation may induce the development of neurological disorders like Parkinson’s disease, Huntington’s disease, and mood disorders (including depression and anxiety) [9]. Growing evidence indicates that environmental neurotoxicants are involved in the development of various forms of neurodegenerative and neurological diseases through trigger epigenetic changes and inducing disruption of the epigenome [10–13]. Some sources of environmental pollutants were related to neurotoxic manifestations, such as metals, pesticides, solvent and some other environmental pollutions [14–16]. Chemicals could regulate gene expression by influencing gene transcription, mRNA degradation and translation, etc. Abnormal changes in DNA or RNA methylation, non-coding RNA, histone modification can serve as biomarkers for environmental pollutant-induced neurotoxicity [17–21]. Consequently, insight into the epigenetic mechanisms by which environmental contaminants causes neurotoxicity is key to modelling targeted preventions and treatments [22].

**Fig. 1:**
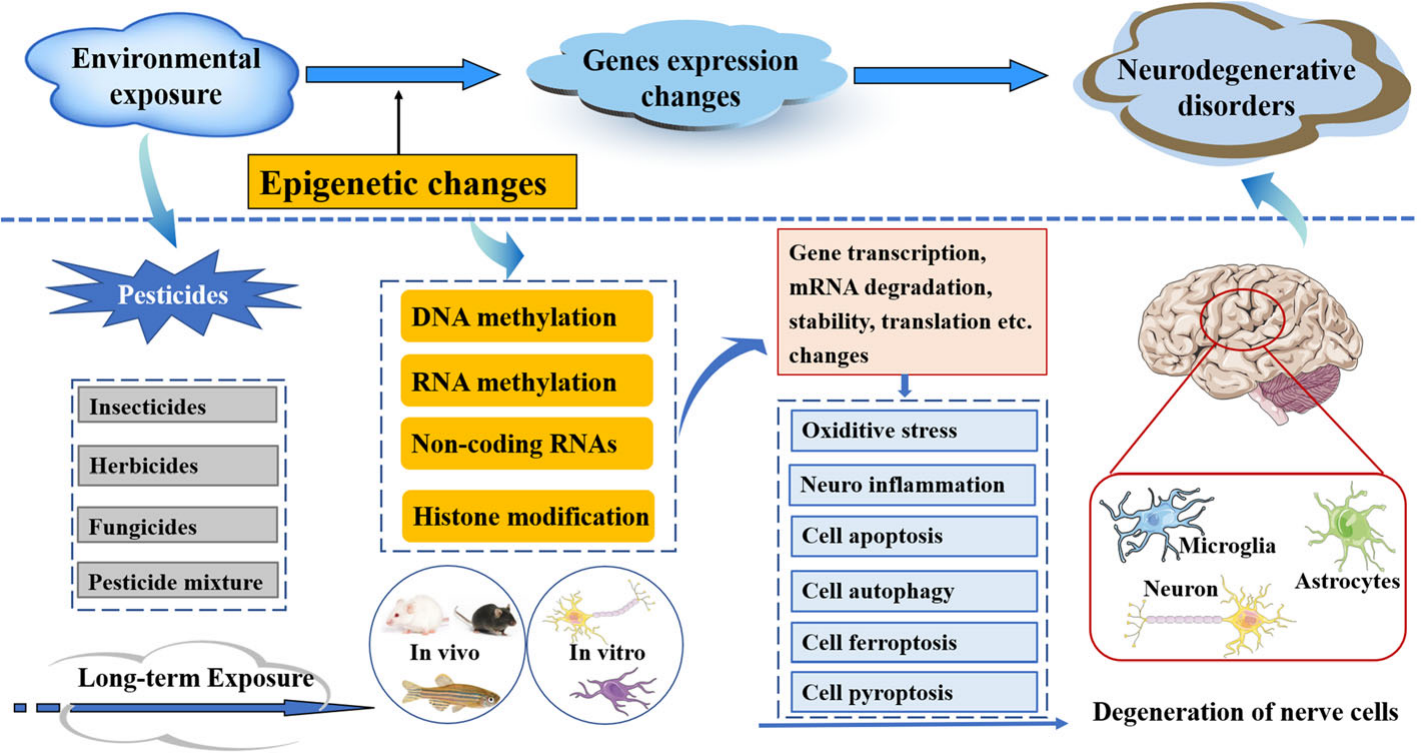
Schematic representation of the mechanism of epigenetic alterations and neurodegenerative disorders due to exposure to different kind of pesticides

Pesticides, including insecticides, herbicides and fungicides, are widely used in agriculture for preventing, destroying, repelling or mitigating harmful or unwanted insects, weeds and fungi. However, most pesticides are not highly selective and generally toxic to many nontarget species, including humans [23]. Particularly, insecticides, which kill insects by disrupting their nervous system, exert neurotoxic effects in humans as well. Neurotoxicity can be induced upon high acute exposure, or by chronic exposure at low doses. Multiple studies have proved chronic exposure to pesticides at a low dose is a risk factor for the development of neurodegenerative diseases, including Alzheimer’s disease, Parkinson’s disease, amyotrophic lateral sclerosis (ALS), and attention deficit hyperactivity disorder (ADHD) etc. [24–30]. However, nowadays there are still about 25 million workers experience unintentional pesticide poisoning each year, due to inhalation or skin absorption [31]. Therefore, it is very important to study the pathogenic mechanism and preventive measures of neurodegenerative and neurological diseases induced by pesticides. For the past few years, many researchers have concerned the potential role of epigenetic mechanism in pesticide induced neurotoxicity (as shown in Fig. 1).

Here we review details studies on epigenetics alterations in nervous systems in relation to pesticides, highlighting potential mechanism in the etiology of various neurodegenerative diseases (summarized Table 1). We also highlight current gaps and future areas for the studies upon epigenetic neurotoxicity induced by pesticides.

**Table 1:** Summary of epigenetic changes induced by pesticisdes

Pesticides	Model	Exposure route	Exposure dose	Exposure length	Neurological damage	Epigenetic change	Ref.
**Insecticides**							
**Chlorpyrifos**	Long Evans rats	Subcutaneous injection	3 or 10 mg/kg	21 days	Associated with problems in cognitive function.	MiR-132 and miR-212 in the CPF-exposed rat hippocampus were up-regulated.	[32]
	SH-SY5Y cells	Cell culture	0, 25, 50, 100, and 200 μM	24 h	Cell viability was decreased. Pyroptosis related proteins, ROS levels, as well as level of caspase-1 and the TUNEL positive cells were all significantly up-regulated.	Expression of miR-181 was enhanced.	[33]
**Chlorpyrifos-oxon**	Zebrafish	Embryonic exposure	0.01 and 50 μg/L	4–120 h post fertilization	Even low dose exposures can have transgenerational effects in neurological activity.	Induce alterations in global DNA methylation.	[34]
**Dieldrin**	C57BL/6 mice	Oral intake	0.3 mg/kg/3 days	30 days	Induce impairment of dopaminergic neuron development and maintenance.	Dieldrin-induced differential methylation was sex-specific.	[35]
	C57BL/6 J mice	Intraperitoneal injection	5.0 mg/kg every other day	30 days	Induce apoptotic cell death in dopaminergic neuronal Cells.	Induce a time-dependent increase in the acetylation of core histones H3 and H4.	[36]
**Permethrin**	Wistar rats	Oral intake	34.05 mg/kg	From PND6 to PND21	Early life permethrin exposure in rats, at a dose close to No Observed Adverse Effect Level (NOAEL) during neonatal brain development leads to its accumulation long after exposure.	DNMT1, DNMT3a were increased. The aggregation of DNMT3b and α-synuclein was enhanced.	[37].
	Wistar rats	Oral intake	34.05 mg/kg	From PND6 to PND21	Low dosage exposure to permethrin during neonatal brain development leads to dopamine decrease in rat striatum nucleus, oxidative stress and behavioural changes linked to the development of Parkinson’s like neurodegeneration later in life.	Global 5mC and 5hmC levels were increased. Methylation levels at H3K9me3 position at both Th and Nurr1 promoter regions were reduced.	[38]
	Wistar rats	Oral intake	34.05 mg/kg	From PND6 to PND21	An intergenerational permethrin-induced damage on progenies has been identified.	Global genome-wide DNA methylation was decreased in mothers exposed in early life to permethrin as well as in their offspring.	[39]
	Wistar rats	Oral intake	34.05 mg/kg	From PND6 to PND21	Parental exposure leads to a significant reduction in dopamine level in the offspring (F1) born from parents or just mothers early-life treated.	Early-life exposure to the stressor is associated with changes in global DNA methylation and hydroxymethylation in adult age.	[40]
**Paraquat**	N27 dopaminergic cells	Cell culture	400 mM	12, 24 or 36 h.	Cell culture models of Parkinson’s disease.	Histone H3 acetylation was induced in a time dependent manner.	[41]
	hNPCs	Cell culture	0, 5, 10, 20, 40, or 80 μM	24 h	Induce developmental neurotoxicity.	Induced differentially miRNAs expression.	[42]
	hNPCs	Cell culture	10 and 100 μM	24 h	Lead to the alteration of several neurodevelopment related key biological processes and crucial pathways.	Alter mRNAs and miRNAs expression.	[43]
	hNPCs	Cell culture	0, 25, 50 and 100 μM	24 h	PQ dramatically suppressed neural cell differentiation ability.	Direct binding effect between CTNNB1 and miR-200a existed following PQ exposure.	[44]
	Neuro-2a cells	Cell culture	100 μM	48 h	CaM and p21 were involved in PQ-induced toxicity.	Nrf2-regulated miR-380-3p inhibited cell proliferation and enhanced the PQ-induced toxicity in N2a cells potentially by blocking the translation Sp3 mRNA.	[45]
	SH-SY5Y cells	Cell culture	50 μM	24 h	Impairs Nrf2/ARE defense network.	Cause miR153 to bind to and target Nrf2 3′ UTR thereby weakening the cellular antioxidant Defense.	[46]
	Neuro-2a cells	Cell culture	300	48 h	Cell culture models of Parkinson’s disease.	The expression of miR-17-5p was downregulated, DNA methylation level was upregulated after PQ exposure.	[17]
	Neuro-2a cells	Cell culture	300	48 h	Induce Parkinson’s disease pathology.	The miR-17-5p was expressed at lower levels in PQ-treated Neuron-2a cells, overexpression of miR17-5p in Neuro-2a cells could enhance cell proliferation, suppresse apoptosis and promote S phase transition of the cell cycle after PQ treatment.	[47]
	ICR mice	Intraperitoneal injection	5, 10 mg/kg/2 days	10 times	Animal models of Parkinson’s disease.	PQ caused lncRNA expression profiling alteration in the substantia nigra (SN) through an interaction with Nrf2, thus changing the NR_027648/Zc3h14/Cybb and NR_030777/Zfp326/Cpne5 mRNA pathways.	[48]
	ICR mice	Intraperitoneal injection	5 mg/kg/3 days, 10 mg/kg/2 days,	10 times	Inhibited microglia and dopaminergic cells proliferation and microglia migration.	PQ-induced low expression of AK039862 rescued microglia proliferation and migration inhibition via the AK039862/Pafah1b1/Foxa1 pathway, AK039862 also participated in the interaction between microglia and dopaminergic cells with PQ treatment.	[49]
	BV2 cells and MN9d cells	Co-culture	0, 50, 100, 150 μM	36 h			
	Neuro-2a cells	Cell culture	100, 300 μM	24, 36 h	Induce neurotoxicity	LncRNA NR_030777 has a vital protective role by regulating the expression of Zfp326 and Cpne 5 in neurotoxicity induced by PQ.	[50]
	MN9D cells						
	Primary cortical neuron						
	Neuro-2a cells	Cell culture	200 μM	3 h	Induce oxidative stress	m^6^A participated in a specific regulatory network of circRNAs to modulate the expression of downstream genes in response to PQ-induced oxidative stress	[51]
**Avermectin**	Pigeons	Oral intake	20, 40, 60 mg/kg	30, 60, 90 days	AVM exhibits significant cytotoxicity in pigeon brain nerve cells.	Global DNA hypomethylation and down-regulation of DNMT mRNA expression occurred in a dose-time-dependent manner in pigeon brains.	[52]
**Fipronil**	Zebrafish	Embryonic exposure	40 μg/L	From 6 to 96 h post fertilization	R-fipronil exhibited more intense neurotoxicity compared with S-fipronil.	The fipronil-conducted enantioselective neurotoxicity likely applied its enantioselectivity by the dysregulation of DNA methylation.	[53]
**Atrazine (ATR)**	Carp	Embryonic exposure	4.28, 42.8 and 428 μg/L	40 days	Not mentioned.	The MBD2 mRNA expression was up-regulated in the brain, the DNMTs mRNA expression was down-regulated	[54]
**Chlorpyrifos (CPF)**			1.16, 11.6 and 116 μg/L				
**Combined ATR/CPF**			1.13, 11.3 and 113 μg/L				
**Deltamethrin**	C57BL/6 N mice	Oral intake	3 mg/kg	During gestation, lactation, and weaning at postnatal day (PND) 21	Deltamethrin insecticide and stress exposure during neurodevelopment leads to alterations in dopamine function (PND21–60).	Hypermethylation of Nr3c1 is in response to dual deltamethrin and corticosterone exposures during development.	[55]
**Corticosterone**		Drinking water	25 μg/mL	From adolescence through adulthood			

## Mechanism of epigenetics

### DNA methylation

DNA methylation is an epigenetic mechanism involving the transfer of a methyl group onto the C5 position of the cytosine to form 5-methylcytosine [56]. Methyl groups to cytosine are added by the DNA methyltransferase (DNMT) enzyme family, namely DNMT3A, DNMT3B, and DNMT1 [57–59]. DNA methylation is the most well studied epigenetic regulators in relation to environmental exposures, which can result in altered global and gene-specific DNA methylation [60]. In the nervous system, neuronal activity can also modulate DNA methylation in response to physiological and environmental stimuli [61, 62].

### RNA methylation

In addition to genomic DNA modifications, various modifications of nucleosides which form the basis for RNA (including tRNA, rRNA, mRNA etc.) were also appreciated [63–65]. N^6^-methyladenosine (m^6^A) in mRNA has been the best-characterized mRNA modification so far, with roles in modulating mRNA transcript processing and regulation [66, 67]. The m^6^A effectors include “writers”, “erasers” and “readers” that respectively install, remove and interpret the methylation [68]. The ‘writer’ methyltransferase enzymes (including METTL3, METTL14, WTAP, and KIAA1429) add a methyl group to the N^6^ position of adenosine in RNA, and removed by the “erasers” demethylases (including FTO and ALKBH5). The methyl group can be recognized by “reader” proteins (HNRNPC, HNRNPA2B1, YTHDF2, YTHDF1, and eIF3), and influence almost all steps of RNA metabolism, including mRNA translation, degradation, splicing, export and folding, consequently altering target gene expression, influencing the corresponding cell processes and physiological function [69, 70].

### Histone modification

Histones (H3, H4, H2A, H2B and H1) are the most abundant proteins in the eukaryotic nuclear DNA structure [71]. Most of the amino acids reside on the N-terminal tails of the histone proteins are subjected to modifications, including acetylation, methylation and ubiquitination on lysine, methylation and citrullination on arginine, etc. [72]. Histone modification can influence all DNA-based processes, including chromatin compaction, nucleosome dynamics, and transcription [73]. Moreover, histone core modifications can also directly regulate transcription, and influence processes of DNA repair, replication, stemness, and changes in cell state [74]. In general, active enhancers are related to the enrichments of both monomethylated H3K4 (H3K4me1) and H3K27ac. Gene bodies of actively transcribed genes are associated with trimethylated H3K36 (H3K36me3), and transcription start sites of actively transcribed genes can be identified by trimethylated H3K4 (H3K4me3) and acetylated H3K27 (H3K27ac) [72].

### Non-coding RNAs

Non-coding RNAs (ncRNA) are a relatively recently described but significant subpopulation of the transcriptome [75]. According to their size, non-coding RNAs can be classified to short RNAs which are < 200 nucleotides (nts) in length and long non-coding RNAs (lncRNAs) are longer than 200 nts. Short non-coding RNAs include PIWI-interacting RNAs (piRNAs), microRNAs (miRNAs), small interfering RNAs (siRNAs), circular RNA (circRNA) and small nuclear RNAs (snRNAs). In the last decade, gene expression has been proved to be largely regulated by ncRNAs. A growing number of studies have investigated the involvement of ncRNAs in various physiological processes [75]. Moreover, miRNAs, lncRNAs and circRNAs have been proved to be involved in transcriptional regulation at different level. Therefore, ncRNA is a burgeoning area for all the biology fields, including neurotoxicology [76].

## Pesticide induced epigenetic neurotoxic effect

### Insecticides

#### Organophosphate pesticide

Organophosphate pesticide has been known as the most widely used pesticides during the past half century. Number of literatures regard their association with neurodegenerative and neurodevelopmental disorders with respect to epigenetic mechanisms [77, 78].

Chlorpyrifos (CPF) is one of the most widely used organophosphorus pesticide (OP) in the world. However, CPE and its active metabolite chlorpyrifos-oxon (CPO) have been proved involving in several neurodevelopmental disorders. Lee’s work identifies the potential epigenetic mechanism of hlorpyrifos neurotoxicity, founding that miR132/212 was elevated in the CA1 hippocampal region, disrupting the neurotrophin mediated cognitive processes after CPF administration [32]. Zhao’s team observed chlorpyrifos could activate cell pyroptosis and increases susceptibility on oxidative stress-induced toxicity by miR-181/SIRT1/PGC-1α/Nrf2 signaling pathway [33]. In addition, the adverse effects of developmental exposure to the active CPO have been proved to persist into adulthood even future generations [79]. Schmitt et al. demonstrated that early life stage exposures to CPO can lead to epigenetic changes in neurological activity, which may lead to alterations in response to CPO in future generations [34]. Liu’ team also investigated H3K4me3 and DNA methylation levels of the PPARγ gene in the placenta was associated with prenatal chlorpyrifos exposure, and could effect on birth outcomes and neurodevelopment [80].

#### Organochlorine pesticide

Organochlorine pesticide is another kind of insecticides. Nowadayes, some OCPs are banned in most industrialized countries. However, due to the very long half-life in humans, the circulating levels of the breakdown product of dchlorodiphenyltrichloroethane (DDT), p,p′-DDE (1, 1-dichloro2, 2-bis (p-chlorophenyl) ethylene) are still found in almost all humans in the industrialized world. Moreover, some OCPs, such as DDT is still used to combat malaria in Asia and Africa [81]. Researches in epidemiology showed workers with Parkinson’s disease were related with exposure to organochlorines [77].

DDT is a common environmental organochlorine pesticide with a long half-life. Because it can passes through the placental barrier, DDT exposure during pregnancy may heavily influence lifetime health of the offspring.. An epidemiological study showed that prenatal exposure to DDT is associated with fetal genome-wide DNA methylation [82]. Another cohort study also investigated prenatal exposure to persistent organic pollutants can induce DNA methylation of LINE-1 and imprint genes in placenta [83].

Dieldrin is also a highly toxic organochlorine pesticide. Although it was phased out of commercial use in the 1970s, dieldrin was still persisted in the environment and easily accumulated in lipid-rich tissues like the brain, due to its high stability and lipophilicity [84]. Previous epidemiology studies have shown a positive association between dieldrin exposure and PD [85, 86]. Experimental researches also investigated that developmental dieldrin exposure could alter DNA methylation at genes related to dopaminergic neuron development and Parkinson’s disease [35]. Song’ research revealed that dieldrin induced a time-dependent increase in the acetylation of core histones H3 and H4 in neuronal cells. Moreover, the histone acetylation appeared within 10 min after exposure to dieldrin, suggesting that acetylation is an early event in dieldrin neurotoxicity [36].

#### Pyrethroid

Pyrethroid insecticides contain natural pyrethrins which are extracted from pyrethrum flowers, and their synthetic derivatives, pyrethroids [87]. Pyrethroids with low mammalian toxicity are now commonly used for household and post-harvest insect control [88]. However, pyrethroid pesticide exposures may be also associated with disruption of neurological functioning [89–92]. Neonatal exposure to permethrin can induce a Parkinson-like disease.

Permethrin is a synthetic pyrethroid widely used as anti-woodworm agent and for indoor and outdoor pest control. However, early life permethrin exposure induces long-term brain changes [93]. These long-term changes were regulated by early impairment of epigenetic pattern in neurodegeneration, such as DNA methylation or histone alterations [37, 38]. In addition, on the prospective intergenerational effect of this pesticide, parental exposure also leads to global DNA methylation changes and hydroxymethylation impairment in their offspring, providing pivotal evidences on intergenerational effects of postnatal exposure to permethrin [39, 40]. Tevoltage-gated sodium channel (VGSC), a motoneuronal transport protein, is the target of all pyrethroids [94]. In addition, Kubik et al. found miRNA-33 modulates permethrin induced toxicity by regulating VGSC transcripts [95].

### Herbicide

**Fig. 2:**
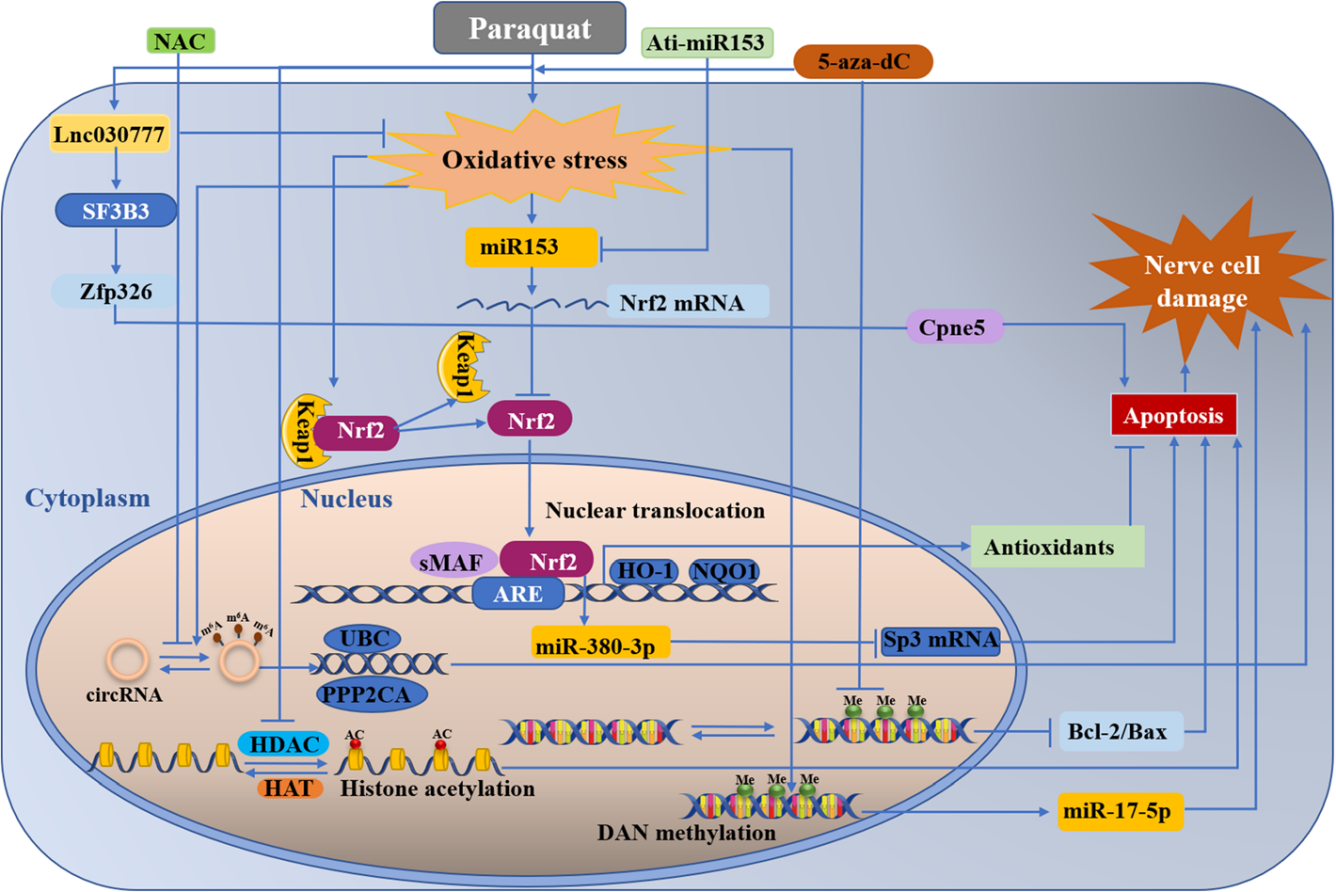
Epigenetic regulation in the context ofparaquat induced nerve cell damage. DAN methylation, m6A modification of circRNA,histone acetylation, miRNA, and lncRNAs were involved in the epigenetic regulation process. The details of the molecular mechanisms were descripted in the following text

Herbicides are essential tool in weed management. The first commercial herbicides were released in the 1940s and hundreds more since then [96]. Among all the herbicides, the neurotoxicity paraquat (PQ) are probably the most conclusive [97]. Paraquat exposure has been linked to an increased risk for Parkinson’s disease [98], and has been used for modeling sporadic Parkinson’s Disease [99]. However, the impacts of PQ exposure on the central nervous system remain unclear. In recent years, epigenetic mechanisms involved paraquat induced neurons damage have been extensively investigated in order to explore new preventive and therapeutic targets of Parkinson’s disease. Our team also do a lot of exploration there [17, 30, 45, 47–51, 100]. The epigenetic molecular mechanisms were summarized in Fig. 2.

As shown in Fig. 2, DNA methylations were found to be causative factors in paraquat induced neurons damage. When pretreated with methyltransferase inhibitor 5′-aza-dC, the level of reactive oxygen species (ROS) increased more significantly. The rate of bcl-2/bax decreased, consequently enhancing the cell apoptosis induced by PQ. This work demonstrated the interaction of DNA methylation and paraquat, providing additional new insights into the pathogenic mechanisms of PD [101].

A major epigenetic change in chromatin can regulate gene expression. Song et al. found that exposure to paraquat could induced histone H3 acetylation in N27 dopaminergic cells in a time dependent manner. These changes were associated with decreased total histone deacetylase (HDAC) activity and HDAC4 and 7 protein expression levels. Additionally, the inhibition of histone acetyltransferase (HAT) activity by anacardic acid can protect against apoptotic cell death induced by PQ, indicating that histone acetylation may represent key epigenetic changes during neurotoxic insults [41].

Evidence indicates that miRNA also play a key role in PQ involved neurodegenerative diseases. Zhou’s team analyzed the impacts of PQ on the miRNome of human neural progenitor cells (hNPCs) during proliferation. Upon PQ treatment, 66 miRNAs were identified as differentially expressed in proliferating hNPCs. With further analysis, the target genes were found enriched in regulation of cell proliferation and differentiation, cell cycle and apoptosis as well as tumor protein 53 (p53), Wnt, Notch and mitogen-activated protein kinases (MAPK) signaling pathways [42]. Furtherly, they found mRNAs and miRNAs expression changes induced by PQ in hNPCs could lead to the alteration of several neurodevelopment related key biological processes and crucial pathways, especially Wnt signaling pathway. The results suggested that PQ could downregulate Wnt signaling pathway via miRNA to induce developmental neurotoxicity [43]. In addition, they also found miR-200a was down regulated in the PQ treated neural stem cell. This process subsequently decreased cell viability, increased epithelial-mesenchymal transition process and the inhibited differential through CTNNB1 pathway [44]. The transcription factor Nrf2 has been proved to regulate the expression of many miRNAs, our previous work also found that Nrf2 can promote the expression of miR-380-3p, which blocks the translation of Sp3 protein and enhances paraquat-induced toxicity in mouse neuroblastoma N2a cells [45]. Interestingly, Narasimhan et al. explained the relationship between Nrf2 and miRNA from a different perspective. Their study showed that PQ induced miR153 dependent hydrogen peroxide (H_2_O_2_), and then caused miR153 to bind to and target Nrf2 3′ UTR thereby weakening the cellular antioxidant defense [46].

Emerging evidence showing that oxidative stress and DNA methylation can alter miRNA expression. Our previous work also investigated that PQ can induce DNA methylation variations through ROS production, leading to the downregulation of miR-17-5p expression [17]. The downregulation of miR-17-5p expression contributes to PQ-induced dopaminergic neurodegeneration through influencing the cell proliferation, cell apoptosis and S phase transition of the cell cycle [47]. Subsequently, we found long noncoding RNAs also play a crucial part in PQ induced neurotoxicity. The alteration of the lncRNA expression profile were found in PQ treated mouse, suggesting lncRNAs were involved in PQ- induced neurotoxicity. Further studies displayed PQ caused lncRNA expression profiling alteration in the substantia nigra (SN) through an interaction with Nrf2, thus changing the NR_027648/Zc3h14/ Cybb and NR_030777/Zfp326/Cpne5 mRNA pathways [48]. Latterly, we proved PQ-induced low expression of AK039862 rescued microglia proliferation and migration inhibition via the AK039862/Pafah1b1/Foxa1 pathway [49]. LncRNA NR_030777 is another lncRNA involved in PQ induced neurotoxicity. It is highly conserved among species and was firstly confirmed by our team. Evidence illustrated NR_030777 7 has a vital protective role in neurotoxicity induced by PQ through regulating the expression of Zfp326 and Copine 5 [50]. These novel discoveries suggested lncRNAs could be a potential target for the prevention and treatment of PQ-induced neurodegenerative disorders such as PD. Recently, our team successfully established a positive link between the alteration of circRNAs driven by m^6^A modification and PQ-induced oxidative stress. Moreover, the alteration of m^6^A methylated circRNAs upon PQ exposure could be partially reversed by N-acetylcysteine (NAC) pretreatment, providing a new preventive or therapeutic tools for PQ-induced neurodegenerative disorders [51].

### Other pesticides

In addition to the pesticides described above, there are still some other pesticides, such as biological insecticides, combination of various pesticides and some other new low toxicity chemical pesticides. The epigenetic mechanisms of their neurotoxicity were also investigated.

#### Avermectin

Avermectin (AVM) is an effective insecticidal and nematicidal agent, it has been extensively used in agriculture and stock farming areas. Subsequently, the residues of AVM or its active metabolites present a toxic threat through epigenetic mechanisms. Cao et al. reported subchronic exposure to AVM could decrease the mRNA expression levels of DNA methyltransferases (DNMT1 and DNMT3a/3b), and increase the mRNA levels of demethylase methyl-CpG-binding domain protein 2 (MBD2), consequently down regulated the global DNA methylation level decreased in pigeon brain tissues [52].

#### Fipronil

Fipronil is a broad-spectrum chiral insecticide. It has been documented to induce significant neurotoxicity to nontarget aquatic species. Qian et al. firstly discussed the enantioselective toxicity of chiral pesticide fipronil in central nervous system, and find R-fipronil exhibited more intense neurotoxicity compared with S-fipronil. Further research revealed that R-fipronil disrupted five signaling pathways including MAPK, Calcium signaling, Neuroactive ligand-receptor interaction, Purine metabolism, and Endocytosis. These pathways revealed greater extent than S-fipronil through the hypermethylation of several important neuro-related genes. This study indicated that the fipronil-conducted enantioselective neurotoxicity by the dysregulation of DNA methylation, providing a novel epigenetic insight into the study of enantioselective biological effects [74].

#### Pesticide mixture

Nowadays, mixed formulation of pesticide is commonly used to reduce insect resistance, achieve-control efficiency and/or economic concers [102]. Xing et al. examined the effect of atrazine (ATR), chlorpyrifos (CPF) and combined ATR/CPF exposure on DNA methylation in the brain of zebra fish. Compared to the control fish, a significant global DNA hypomethylation was observed in the common carp exposed to ATR, CPF and their mixture. Moreover, the MBD2 mRNA expression was up-regulated and the DNMTs mRNA expression was down-regulated in the brain and gonad of the common carp exposed to ATR, CPF and their mixture [75]. Nuclear receptor subfamily 3 group C member 1 (Nr3c1) is a transcription factors necessary for proper dopamine neuron development. Vester et al. also reported combined neurodevelopmental exposure to deltamethrin and corticosterone could induce Nr3c1 hypermethylation in the midbrain of adult male mice [76].

## Concluding remarks

The underlying cause of many neurological and neurodegenerative diseases are still unclear. However, it is now widely accepted that the environmental toxicants contribute to many of these disorders. Increasing evidence indicates that the epigenome may be targeted on people at risk for neurodegenerative disorders. Multiple studies have indicated chronic exposure to pesticides at a low dose could increase the risk of neurodegenerative diseases. Epigenetic disruptions induced by pesticides, which can induce neurotoxicity were summarized in this work. Epidemiologic studies and experimental researches performed both in vivo and in vitro provide evidence that exposure to pesticides even at a low dose have long-term effects on central nervous systems, possibly via epigenetic modifications. Individuals, exposure to neurotoxic in the early life and even in the womb could induce epigenetic alterations in adulthood, the aged or even next generations, moreover some of the alterations are sex-depended. Meanwhile, compared with genetic changes, epigenetic aberrations could be more easily reversible. Drugs, even some nutrients, which target the specific epigenetic mechanisms involved in the regulation of gene expression could be emerging preventive or therapeutic tools for disease. In this review, there are some evidences illustrated inhibits the epigenetic modification changes by compounds or regulates the expression of non-coding RNA by genetic tools might alleviate pesticide induced neurological and neurodegenerative impairments. These will shed new light on the precise prevention and targeted treatment for the neurodegenerative diseases. However, a great deal of further investigations is still needed. For instance (1) explore how pesticides exposure leads to epigenetic alteration in specific genes; (2) identify the susceptible genes of human in the epigenetic alterations induced by exposure to pesticides; (3) prove whether epigenetic changes can be used as biomarkers for the early detection and treatment target for neurodegenerative disorders; (4) develop novel epigenetic modification inhibitors; (5) explore the epigenetic mechanisms of some novel and mixtures of pesticides.

## Data Availability

Not applicable.

## Abbreviations

AD: Alzheimer’s disease
ADHD: Attention deficit/hyperactivity disorder
ALS: Amyotrophic lateral sclerosis
AVM: Avermectin
circRNA: Circular RNA
CNS: Central nervous system
CPF: Chlorpyrifos
CPO: Chlorpyrifos-oxon
DDT: Dchlorodiphenyltrichloroethane
DNMT: DNA methyltransferase
HAT: Histone acetyltransferases
HDACs: Histone deacetylases
hNPCs: Human neural progenitor cells
lncRNAs: Long non-coding RNAs
m^6^A: N6-methyladenosine
MAPK: Mitogen-activated protein kinases
MBD2: Methyl-CpG-binding domain protein 2
miRNA: Micrornas
ncRNA: Non-coding RNAs
NAC: N-acetylcysteine
OP: Organophosphorus pesticide
PD: Parkinson’s disease,
piRNAs: PIWI-interacting RNAs
PQ: Paraquat
ROS: Reactive oxygen species
siRNAs: Small interfering RNAs
SN: Substantia nigra
snRNAs: Small nuclear RNAs
